# Insomnia in Schizophrenia Patients: Prevalence and Quality of Life

**DOI:** 10.3390/ijerph17041350

**Published:** 2020-02-19

**Authors:** David Batalla-Martín, Angel Belzunegui-Eraso, Eva Miralles Garijo, Elena Martínez Martín, Rosanna Romaní Garcia, Jacobo San Miguel Heras, Marina Lopez-Ruiz, Maria Antonia Martorell-Poveda

**Affiliations:** 1 Nou Barris Mental Health Center, 08016 Barcelona, Spainelena.martinez@csm9b.cat (E.M.M.); rosanna.romani@csm9b.com (R.R.G.); jacobo.sanmiguel@csm9b.com (J.S.M.H.); 2 Social Inclusion Chair, Rovira i Virgili University, 43002 Tarragona, Spain; angel.belzunegui@urv.cat; 3 Service of Psychiatry and Psychology, HM-Sant Jordi Clinic, 08030 Barcelona, Spain; marinalopezr@gmail.com; 4 Nursing Department, Faculty of Nursing, Rovira and Virgili University, 43002 Tarragona, Spain; mariaantonia.martorell@urv.cat

**Keywords:** insomnia, schizophrenia, sleep disturbance, quality of life, Oviedo Sleep Questionnaire, Insomnia Severity Index, prevalence, comorbidity, mental health

## Abstract

Sleep disorders are often not regarded as an important health problem, despite their impact on heath. Insomnia is the most frequent sleep disorder in mental health. The aim is to quantify the prevalence of insomnia in a population with schizophrenic disorder and assess its influence on quality of life. This is a descriptive, analytical and cross-sectional study conducted in a sample of 267 schizophrenic patients over 18 years of age using consecutive non-probabilistic sampling. The variables of interest were collected by means of the "Cuestionario Oviedo de Sueño," "Insomnia Severity Index" and EuqoQol-5D. The estimation of insomnia in our schizophrenic population according to the International Classification of Disease (ICD-10) criteria was 23.2%. The likelihood of insomnia when there are problems in the quality of life is significant in all its dimensions: mobility OR: 3.54 (95% CI 1.88– 6.65), self-care OR: 2.69 (95% CI 1.36–5.32), usual activities OR: 3.56 (95% CI 1.97–6.44), pain/discomfort OR: 4.29 (95% CI 2.37–7.74) and anxiety/depression OR: 3.01 (95% CI 1.61–5.65). The prevalence of insomnia fluctuates depending on the diagnostic criteria; however, the schizophrenic population shows high prevalence in some clinical characteristics. People with insomnia have a lower quality of life.

## 1. Introduction

Sleep is an unconscious physiological state characterized by a temporary cessation of sensory activity, mobility and alertness. The need for sleep is biological and occurs periodically, in cycles, and it urges the body to get the rest it needs to replenish the energy expended during the daytime phase. Sleep is an active state during which changes in bodily functions and mental activities take place that have a substantial impact on an individual’s physical and psychological equilibrium [1]. Although most people spend one-third of their life sleeping, sleep is a phenomenon whose mechanisms and functions are not yet fully understood [2].

Insomnia is the most common sleep disorder among people with psychiatric disorders [3] and the most prevalent ailment in the general population, suffered at some point in life by 40% of the population [4]. The prevalence figures for insomnia vary greatly from study to study, primarily due to methodological differences related to the definition used and the diagnostic method applied [5].

These differences are reflected in the review published by Ohayon [6] in 2002. In 50 epidemiological studies conducted using representative samples of the community, the data showed that at least 33% of the population presented some manifestation of insomnia. However, the prevalence decreased to between 16% and 21% when the criterion of frequency of clinical manifestations was included or if only the diagnostic criteria of the DSM-IV was taken into consideration, which reduced the prevalence to a range between 4.4% and 6.4% of the population studied.

Zhang and Wing [7] conducted a meta-analysis in 2006 that examined sex differences in the prevalence of insomnia using 31 studies. They found that women had a 1.41 (95% CI, 1.28–1.55) higher risk ratio than men for insomnia, similar to that observed by Fritsch Montero et al. (8), who concluded that being a woman has an OR of 1.43 for insomnia. 

As the prevalence of insomnia in the general population in Spain has been the focus of little research [8,9], very little data is available on the subject. In 2010, Ohayon and Sagales [10] published a study conducted by means of telephone interviews with 4065 people from all over Spain. They found that insomnia affects 6.4% (95% CI, 5.6%–7.1%) of the Spanish population according to the DSM-IV criteria. However, as little data as there is available for the general population, for the population with schizophrenic disorder, even less information has been published about the prevalence of insomnia. Although insomnia has been studied among hospital patients [11,12,13,14] in conjunction with different comorbidities, to our knowledge no studies have been undertaken in the Spanish outpatient population.

International studies on insomnia among the population with severe mental disorders, and specifically among people with schizophrenic disorder who undergo follow-up and monitoring at outpatient centers, report varying results depending on the diagnostic criteria applied and the diagnostic tool used, either the most frequently used manuals, such as the ICD-10, DSM-IV or ICSD-2, or the most current, like the DSM-V or ICSD-3 [15,16,17,18,19,20].

The health effects of insomnia have also been minimally studied from an epidemiological perspective, with the exception of its relationship to psychopathology or in genetic studies that were not conclusive [21,22]. However, several studies have found a connection between insomnia and a poor general state of health and a worse self-perception of health [23,24,25,26].

Sleep disorders, and especially insomnia, can be a major risk factor for somatic and psychological disorders and can negatively affect quality of life. Certain sleep-related pathologies have been found to be major risk factors for health, regardless of aspects such as age, sex, obesity or tobacco use [27]. Therefore, people who report insomnia have a higher likelihood of morbidity and mortality [28]. 

Zeithlhofer et al. [25] analyzed quality of sleep and quality of life in a total of 1049 Austrian adults over 15 years of age in the general population. The authors concluded that these two variables are closely related. Therefore, poor sleep quality can be a good predictor of quality of life and can be used as a screening indicator in the exploration of people’s quality of life. 

The impact of insomnia on health-related quality of life (HRQOL) can be considerable [29], affecting various different aspects of life, such as the ability to perform everyday tasks and participate in social activities [28]. Patients suffering from chronic insomnia not only have problems that affect their health but also their ability to function in social and employment settings. They frequently complain of emotional, cognitive and behavioral symptoms and detrimental effects on their performance in social and work environments, for example, due to increased absenteeism [30,31].

The aim of this study is to establish the prevalence of insomnia using the diagnostic criteria of the ICD-10 and the DSM-IV and to determine its relationship with HRQOL in a sample of patients diagnosed with schizophrenia who receive outpatient follow-up care and monitoring at the Nou Barris Mental Health Center in Barcelona.

## 2. Materials and Methods

### 2.1. Subjects

Our sample was made up of 267 patients. The sample size was calculated based on an estimated proportion of insomnia of 50%, an accepted error rate of 5% and a confidence level of 95%. The subjects were selected by means of non-probability sampling between February 2019 and April 2019 using the following inclusion criteria: adult men and women with a diagnosis of 295.xx according to the DSM-IV or F20–F29 according to the ICD-10 who were users of the Nou Barris Mental Health Centre, and who maintained the ability to read and write. The exclusion criteria were: presentation of terminal organic pathology, lack of knowledge of the Catalan or Spanish language, illiteracy, diagnosis of mild or severe mental retardation, neurological disease involving cognitive impairment or clinical decompensation of schizophrenic disorder. 

### 2.2. Instruments

Subjects responded to a protocol that included sociodemographic factors, anthropometric measurements, the Oviedo Sleep Questionnaire (OSQ), the Insomnia Severity Index (ISI) and the EuroQol-5D scale (EQ-5D).

#### 2.2.1. Oviedo Sleep Questionnaire (OSQ)

The presence of insomnia was evaluated by means of the Oviedo Sleep Questionnaire (Figure S1) [32,33], an instrument designed to aid in the diagnosis of insomnia-type sleep disorders based on the criteria of the DSM-IV and the ICD-10. Bobes et al. [32] validated this scale in users with depression and obtained a Cronbach’s alpha coefficient for reliability of 0.76. The instrument also showed adequate concurrent validity in comparison with the Hamilton scale (Pearson’s r of 0.78). Subsequently, Garcia-Portilla et al. [34] examined the reliability and validity of the OSQ in patients with severe mental disorders with an internal consistency of 0.91 for the items making up the insomnia scale. The consistency value for the total OSQ was 0.90. The Pearson coefficient for test-retest reliability was 0.87. The OSQ has therefore been shown to have good psychometric performance in patients with schizophrenic disorder. 

#### 2.2.2. Insomnia Severity Index (ISI)

Insomnia severity was assessed using the Insomnia Severity Index (ISI) [35], a self-applied instrument designed to briefly assess the severity of insomnia in the general population based on the diagnostic criteria of the DSM-IV and the International Classification of Sleep Disorders. This scale has been found to have adequate psychometric properties in studies conducted using the English version [36,37], with internal reliability values (Cronbach’s *a*) between 0.74 and 0.90, and test-retest reliability equal to 0.89 one month after evaluation, 0.77 two months after, and 0.73 three months after. Two validation studies of the Spanish version of the ISI were found: one conducted by Fernandez-Mendosa et al. in a young adult population and a middle-aged population [38] in which the factorial model was replicated and showed an internal consistency of 0.82, and one conducted by Sierra et al. with an elderly population [39] in which an internal consistency reliability of 0.91 was found. It has also shown the ability to differentiate between men and women, people with and without cognitive impairment and people with and without medical treatment [40]. 

#### 2.2.3. EuroQol-5D (EQ-5D)

Health-related quality of life (HRQoL) has been assessed using the EuroQol-5D Scale (EQ-5D) [41,42], a self-applied scale that is quick and easy to administer, yielding a multidimensional description of general health as well as a numerical health profile. The scale is made up of two parts, the EQ-5D descriptive system and the visual analogue scale (EQ-VAS). This scale has been validated in Spain by Xavier Badia [43,44]. For psychometric properties, the scale presented a test-retest reliability between 0.86 and 0.90 [45] and a strong correlation with the SF-36 scale [46]. The EQ-5D scale has also been shown to be valid for use with patients diagnosed with schizophrenia [47]. 

### 2.3. Design and Procedure

We conducted a descriptive, analytical and cross-sectional study that aimed to understand the prevalence of insomnia and its association with quality of life in patients diagnosed with schizophrenia. The interviews were conducted by each patient’s nursing team in individual sessions at the mental health centre. The study rigorously followed international ethical recommendations for medical research and was conducted under the ethical principles stipulated in the Declaration of Helsinki. The participants were also informed that all data would be treated as confidential in accordance with regulation (EU) no. 2016/679 and Spanish Organic Law 3/2018 of 5 December on the protection of personal data and the guarantee of digital rights. The study received prior approval from the Ethics Committee of the Unió Catalana de Hospitals (UCH) with registration code CEI 19/10.

### 2.4. Data Analysis

A descriptive analysis was performed based on measures of the central tendency and dispersion of quantitative variables and the absolute and relative frequencies for categorical variables. We used the Student’s t-test to compare means and the χ² test to study the association between nominal and ordinal variables. Pearson’s r, Cramer’s V, Cohen’s d and Yule’s Q coefficients were used to determine the correlation between variables and the magnitude of the effect. Finally, a logistic regression analysis was performed to evaluate the association of insomnia with the study variables. The dimensions of the EQ-5D scale were dichotomised for presence of problem/absence of problem to calculate the odds ratio and estimate the logistic regression. All analyses were considered significant when *p* < 0.05 and were performed using the SPSS v20 statistical package.

## 3. Results

The prevalence of insomnia was 23.2% (95% CI, 18.5%–28.6%) according to the ICD-10 criteria, and 7.9% (95% CI, 5.2%–11.7%) according to the DSM-IV diagnostic criteria. The prevalence of the presence of any of the clinical symptoms of insomnia was 63.7% (95% CI, 58.24%–69.76%). Statistically significant differences were found based on sociodemographic factors among the subgroups with and without insomnia. Insomnia was directly associated with age (*t* = −2.978; *p* = 0.003; *d* = 0.432), educational attainment (no primary education, primary education, secondary education, university education) (*χ²* = 10.490; *p* = 0.015) and body mass index (BMI) (*t* = −2.685; *p* = 0.008; *d* = 0.389). The use of drugs in the study sample was not shown to have a significant statistical relationship with insomnia. The comparison of subgroups as they relate to the set of sociodemographic variables is presented in Table 1.

The prevalence of insomnia according to the ISI was 41.2% (95% CI, 35.30%–47.10%). By severity subgroups, the prevalence of mild insomnia was 27.7% (95% CI, 22.33%–33.07%), the prevalence of moderate insomnia was 10.5% (95% CI, 6.8%–14.18%) and severe insomnia was 3% (95% CI 0.95%–5.1%).

A positive relationship was found between the severity of insomnia according to the diagnostic criteria of the OSQ and the presence of insomnia according to the criteria of the ISI (*p* = 0.000). We observed a strong correlation in the association of the two scales in relation to the severity of insomnia (r = 0.825).

Statistically significant differences were found in the association of health problems according to the EQ-5D dimensions and the clinical presence of insomnia (Table 2). The visual scale of the EQ-5D also shows differences between subgroups with insomnia and without insomnia (*t* = 6.926; *p* = 0.000; *d* = 1.004).

Logistic regression also revealed that the presence of insomnia increases the likelihood of having problems with mobility (OR: 3.54, 95% CI, 1.88–6.65), with self-care (OR: 2.69, 95% CI, 1.36–5.32), with usual activities (OR: 3.56, 95% CI, 1.97–6.44), with pain/discomfort (OR: 4.29, 95% CI 2.37–7.74) and with anxiety/depression (OR: 3.01, 95% CI 1.61–5.65) (Table 3). 

## 4. Discussion

The diagnostic variability of insomnia makes it difficult to compare the results of different studies. For this reason, we analysed insomnia based on different diagnostic instruments. In our study, the prevalence of insomnia according to the DSM-IV criteria was 7.9%, similar to the prevalence observed in other studies with general population samples. Ohayon [6] detected a prevalence among the general population of between 4.4% and 6.4%, and in two other American studies, Ancoli-Israel et al. [48] and Bixler et al. [49] also detected a similar presence of the disorder, specifically 6% and 7.5%, respectively. 

In our study, the prevalence of insomnia in patients diagnosed with schizophrenia was no higher than that found in other studies conducted in the general population when the pathology was diagnosed using the DSM-IV manual, but a higher prevalence was found according to the ICD-10 criteria. The frequency of symptoms is one of the criteria for the diagnosis of insomnia, and the difference between the two manuals in this regard explains these differences. However, if we look at the outcome obtained by Seow et al. [20] using the DSM-V, they detected a prevalence of insomnia similar to the one we found in our research, 23.2% with the ICD-10 and 25% with the DSM-V. We think this similarity can be explained by the modification of the diagnostic criteria between the DSM-IV and the DSM-V in relation to the number of nights/week suffering sleep problems, which has decreased three times a week in the new DSM-V manual. Furthermore, as described by Chung et al. [50], as long as there is neither enough nor better data based on the DSM-V and ICSD-3 criteria, the results studied according to the DSM-IV-TR, ICD-10 and RDC/ICSD-2 remain important. In our opinion, this comparative analysis should be a line of future research.

Other studies conducted with samples of patients with schizophrenia have based their diagnosis of insomnia on the results of the ISI or on other criteria, such as the study by Hou et al. [16], which, after administering three diagnostic questions, concluded that insomnia was prevalent in 28.9% of the sample studied. Mondal et al. [18], using the ISI, observed the presence of moderate to severe insomnia in 29.2% of the sample analysed, a much higher percentage than the 13.5% obtained with the same instrument in our study. Our results are in greater agreement with the presence of insomnia found by Darchia et al. who detected a prevalence of moderate to severe insomnia of 11.2%.

Some of the diagnostic criteria for insomnia in patients with schizophrenia were present in higher proportions than in the general Spanish population. According to Ohayon and Sagales [10], 22.8% (95% CI, 19.6%–22.1%) of the population presented some symptoms, which is similar to that observed by Vela-Bueno et al. [8] in the community of Madrid, where 20.8% of the sample was reported to have symptoms of insomnia. These percentages were lower than those detected in our sample, 63.7% of whom presented some symptoms. 

In contrast, the presence of symptoms did yield a result consistent with those observed in other studies of subjects with schizophrenia. According to an analysis carried out by Cohrs [51], sleep disorders are present in a percentage band ranging from 30% to 80% of people with schizophrenia. Kaufmann et al. [17] placed the figure at 78%, and in the study by Mondal et al. [18], sleep problems affected 83.4% of the sample. These data, added to that observed in this study, emphasise the need to focus attention on all of the diagnostic criteria.

Insomnia’s link to the various sociodemographic factors studied is evidenced in the significant differences detected between the presence of sleep disorder and age (*p* = 0.003). However, our results differ with those of other studies with regard to sex. We did not find significant differences based on sex in our sample (*p* = 0.069) unlike Zang & Wing [7] in their meta-analysis or Fritsch Montero et al. [52]. Nevertheless, the presence of insomnia was still higher in women (28.97%) than in men (19.38%). 

Age is also a factor that affects the presence of insomnia, as observed by Zang and Wing [7]. This was something we corroborated with our results, in this case, in a population with schizophrenic disorder who had a statistically significant higher average age (*p* = 0.003) than that of people who do not suffer from insomnia. An analysis by age groups also demonstrates that the presence of the disorder increases as age increases. 

Other variables found to have a statistically significant relationship with insomnia are educational attainment (*p* = 0.015) and body mass index (BMI) (*p* = 0.008). The association of BMI with the presence of insomnia is in agreement with the results published by Miró et al. [27] who linked insomnia to obesity, thus representing a double risk factor for health.

We explored several dimensions of health-related quality of life and found a statistically significant relationship between those dimensions and insomnia. We can therefore confirm that the presence of insomnia leads to a decrease in health-related quality of life. 

Our data are congruent with those of Taylor et al. [23,53] who found an association between insomnia and poorer general health and negative self-perception of health. In addition, according to Taylor et al., anxiety and depression are dimensions that worsen in the presence of insomnia, and, at the same time, are predictors of chronic insomnia. In the population with schizophrenic disorder, our results also indicate a higher likelihood of insomnia if symptoms of anxiety and depression are also present. 

Taylor et al. and Ford et al. [54,55] associated the presence of pain, sometimes non-specific pain, with the presence of insomnia and even suggested that the presence of insomnia increases somatic complaints of pain. The results obtained from our sample indicate that individuals in pain are 4.29 times more likely to experience insomnia.

Another factor associated with the presence of insomnia is the difficulty in carrying out day-to-day activities; in individuals with insomnia the likelihood of presenting problems in this regard increases considerably. These results are consistent with those analysed by Roth et al. and with the findings of Leger and Poursain [28,31], who conclude that insomnia affects the performance of daily tasks, decreases participation in social activities and increases work-related impairments. 

### 4.1. Study Limitations

This study has various strengths that should be highlighted. Firstly, it is the first study to look at the prevalence of insomnia in outpatient mental health patients with schizophrenic disorder and analyse the relationship between this sleep disorder and health-related quality of life. Secondly, the sample (n = 267) is representative of the population served by the health centre itself. Thirdly, the prevalence and diagnosis of insomnia were determined in accordance with the definitions and symptoms described in the clinical diagnostic manuals, a rare occurrence, as many other studies have based their prevalence results on scales that are not governed by the accuracy of the diagnostic criteria. 

However, this study also has some limitations. This is a cross-sectional study with a sample from a single mental health centre and the statistical inference only corresponds to the population of that health centre with the same pathology. Nevertheless, the study was conducted using a methodology similar to that used in other studies and can be reproduced at other centres in order to compare results. In addition, the sample was not randomly selected, but rather we made use of non-probabilistic consecutive sampling which is considered the best non-probabilistic sample type because it includes all the available subjects. Another limitation of this investigation is we did not use the diagnostic criteria of the newest diagnostic manuals, such as the DSM-V or the ICSD-3, because of the lack of validated diagnostic questionnaires for this purpose. Finally, we believe it is important to highlight the possible relation between insomnia and other sleep disorders in the schizophrenic disorder population [56], an analysis that has not been included in our study and would be very interesting to consider in future research.

### 4.2. Future Implications

Focusing our efforts on decreasing the presence of problems in the affected dimensions of quality of life can decrease the prevalence of insomnia. We believe that adapting a behavioural group intervention programme to focus our interventions on the variables that have a statistical relationship with insomnia would improve quality of life and reduce the prevalence of this sleep disorder and its repercussions on health.

## 5. Conclusions

Our study did not reveal remarkable differences in the prevalence of insomnia among the population with schizophrenic disorder compared to the general population, but we did find that insomnia substantially influences the health-related quality of life in the studied population. The data on the prevalence of insomnia vary depending on the diagnostic manual and the diagnostic tool used, which makes it difficult to obtain definitive data. Some of the diagnostic symptoms present a very high prevalence in this population. Insomnia is a major public health issue, especially in this group. The use of different strategies to minimise the presence of sleep disorders should be a priority in care plans, which will result in improved quality of life. 

## Figures and Tables

**Table 1:** Sociodemographic and clinical variables according to presence/absence of insomnia

	Insomnia (n = 62)		No insomnia (n = 205)		χ²/*t*	*p*
	n	%	n	%		
*Sex*					3.312	0.069
Male	31	19.38	129	80.62%		
Female	31	28.97	76	71.03		
*Age (mean ± SD* *)*	52.98 (± 12.58)		47.87 (± 12.19)		−2.978	0.003
*Age*					13.345	0.020
17–26	1	7.69	12	92.31		
27–35	2	7.14	26	92.86		
36–45	10	18.87	43	81.13		
46–55	27	28.72	67	71.28		
56–65	13	22.03	46	77.97		
Over 66	9	45.00	11	55.00		
*Marital status*					4.084	0.252
Single	27	18.49	119	81.51		
Married / In relationship	19	29.69	45	70.31		
Separated / Divorced	14	28.00	36	72.00		
Widowed	2	28.57	5	71.43		
*Educational attainment*					10.490	0.015
No primary school	9	37.50	15	62.50		
Primary school	36	29.03	88	70.97		
Secondary school	14	13.86	87	86.14		
University	3	16.67	15	83.33		
Employment status					7.026	0.219
*Special work centre*	1	11.11	8	88.89		
Freelancer	2	50.00	2	50.00		
Salaried employee	3	13.04	20	86.96		
Unemployed	5	17.24	24	82.76		
Disability	42	23.60	136	76.40		
Retired	9	37.50	15	62.50		
*Degree of disability* (mean ± SD)	58.13 (± 23.01)		56.54 (± 23.46)		−0.508	0.612
*Link to resources*					3.562	0.313
No link	43	25.00	129	75.00		
SRC	9	20.45	35	79.55		
Pre-employment	1	5.88	16	94.12		
Social club	9	26.47	25	73.53		
*Income level*					2.528	0.470
No income	6	22.22	21	77.78		
Less than minimum wage	37	24.18	116	75.82		
Minimum wage	1	6.67	14	93.33		
More than minimum wage	18	25.00	54	75.00		
*BMI* *(mean ± SD)*	30.75 (± 5.48)		28.60 (± 5.54)		−2.685	0.008
*BMI*					6.186	0.045
Normal weight	13	17.57	61	82.43		
Overweight	16	18.39	71	81.61		
Obese	33	31.13	73	68.87		
*Antipsychotics*					1.228	0.268
Yes	62	23.57	205	76.78		
No	0	0	0	0		
*Antidepressant*					3.505	0.061
Yes	25	30.49	57	69.51		
No	37	20.00	148	80.00		
*Mood stabilizer*					1.700	0.192
Yes	3	12.50	21	87.50		
No	59	24.28	184	75.72		
*Anxiolytics*					0.560	0.454
Yes	33	25.19	98	74.81		
No	29	21.32	107	78.68		

**Table 2:** EuroQol-5D Scale (EQ-5D) dimensions according to presence/absence of insomnia

	Insomnia		No insomnia		χ²/*t*	*p*
	n	%	n	%		
*Mobility*					17.281	0.000
No problems	38	17.92	174	82.08		
Moderate problems	24	44.44	30	55.56		
Severe problems	0	0.00	1	100.00		
*Self-care*					8.664	0.013
No problems	44	19.82	178	80.18		
Moderate problems	17	39.53	26	60.47		
Severe problems	1	50.00	1	50.00		
*Usual activities*					18.918	0.000
No problems	24	14.46	142	85.54		
Moderate problems	36	37.50	60	62.50		
Severe problems	2	40.00	3	60.00		
*Pain/Discomfort*					31.693	0.000
No problems	22	13.25	144	86.75		
Moderate problems	30	34.88	56	65.12		
Severe problems	10	66.67	5	33.33		
*Anxiety/Depression*					23.461	0.000
No problems	16	13.22	105	86.78		
Moderate problems	33	26.61	91	73.39		
Severe problems	13	59.09	9	40.91		
*Evolution past year*					25.166	0.000
Better	21	14.38	125	85.62		
No change	25	26.88	68	73.12		
Worse	16	57.14	12	42.86		
*EQ-VAS (mean ± SD)*	52.37 (± 19.33)		69.79 (± 16.70)		6.926	0.000

**Table 3:** Logistic regression analysis.

	n (% with Problems)			Confidence Interval 95%				
	Insomnia	No Insomnia	Odds Ratio	Lower Limit	Upper Limit	Likelihood	*p*	Yule’s Q
Mobility	24 (38.7%)	31 (15.1%)	3.54	1.88	6.65	0.78	0.000	0.561
Self-care	18 (29%)	27 (13.2%)	2.69	1.36	5.32	0.72	0.004	0.459
Usual activities	38 (61.3%)	63 (30.7%)	3.56	1.97	6.44	0.78	0.000	0.562
Pain/Discomfort	40 (64.5%)	61 (29.8%)	4.29	2.37	7.74	0.81	0.000	0.622
Anxiety/Depression	46 (74.6%)	100 (48.8%)	3.01	1.61	5.65	0.75	0.001	0.502

## Supplementary Materials

The following are available online at https://www.mdpi.com/1660-4601/17/4/1350/s1, Figure S1: Cuestionario Oviedo del Sueño.
