# Supplementary figures and images for: Insomnia in Schizophrenia Patients: Prevalence and Quality of Life
Source: Int J Environ Res Public Health. 2020 Feb 19;17(4):1350. doi: 10.3390/ijerph17041350 (PMC7068578; doi:10.3390/ijerph17041350)

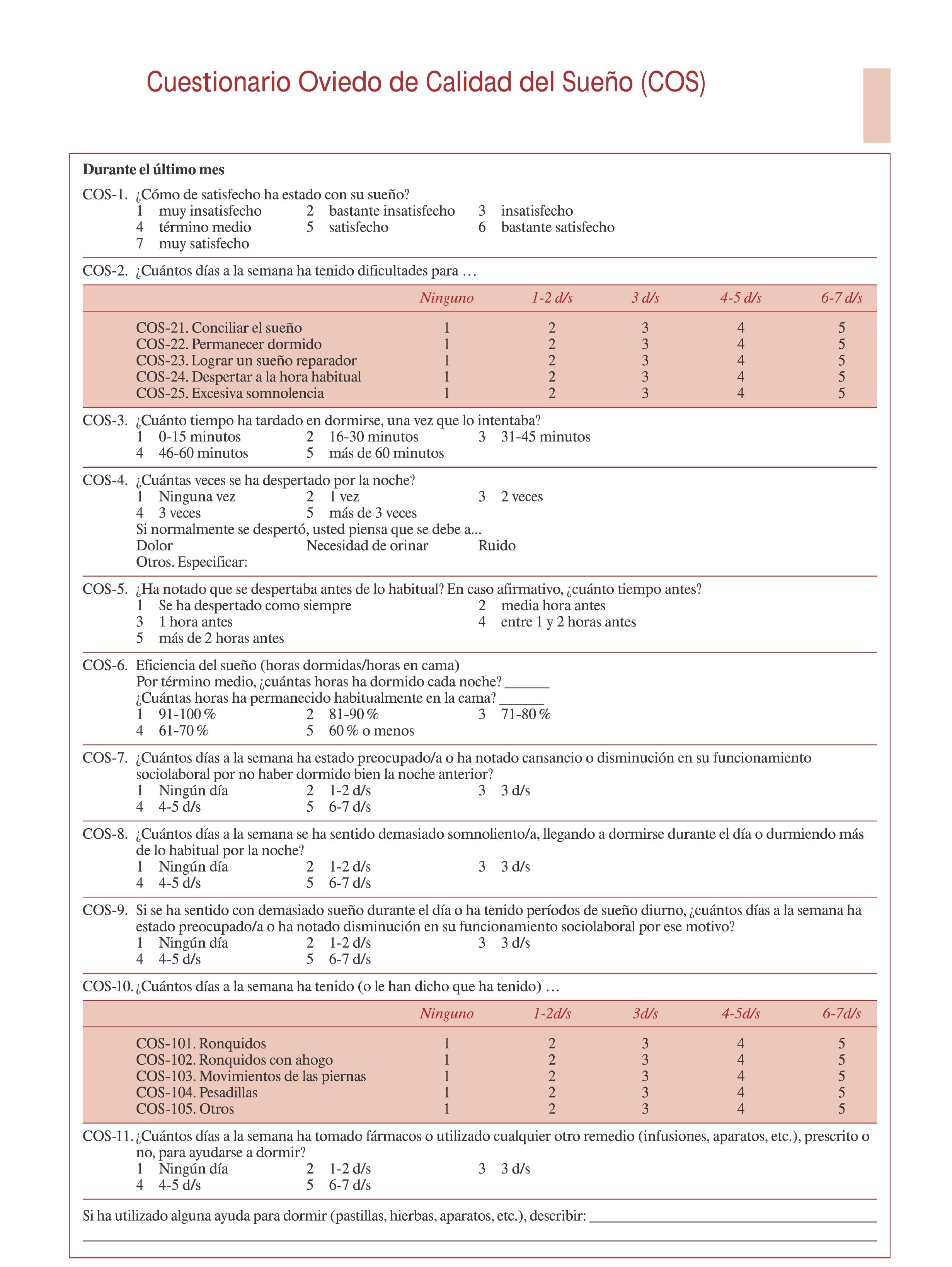

Supplement: Supplementary file 1 [file ijerph-17-01350-s001.zip › ijerph-717119-supplementary.jpg]
